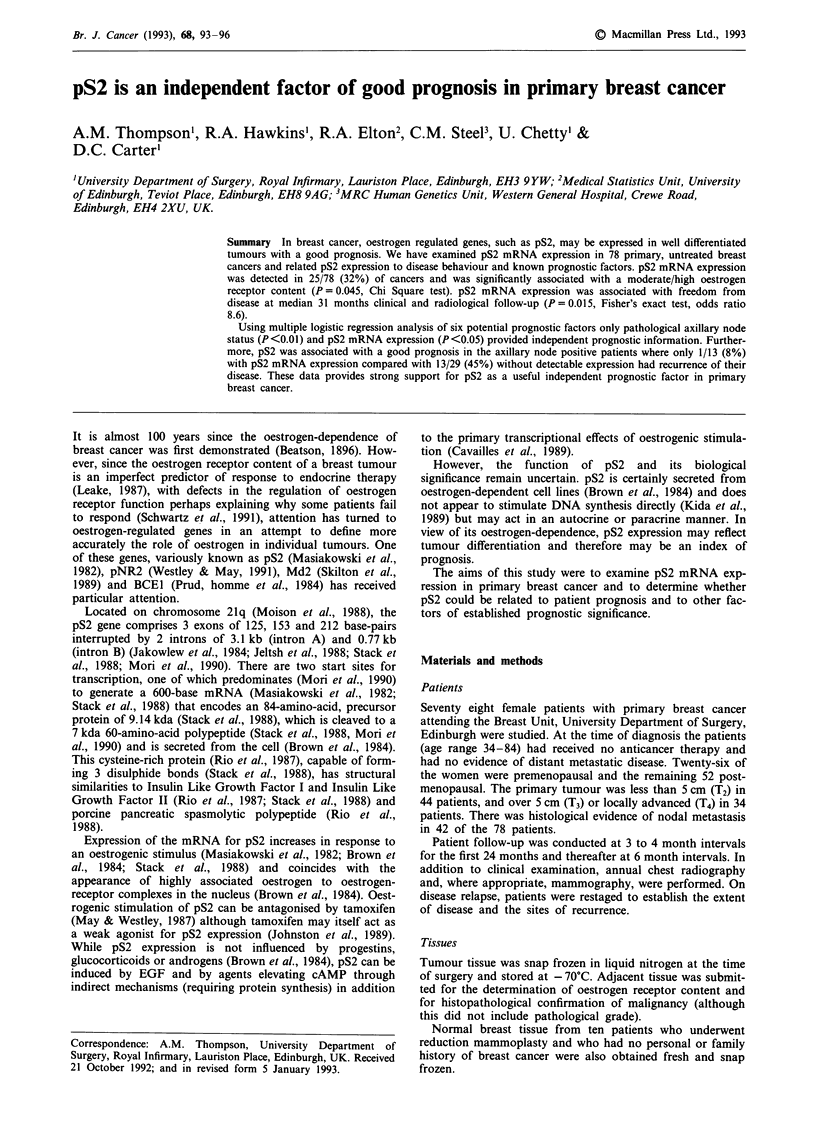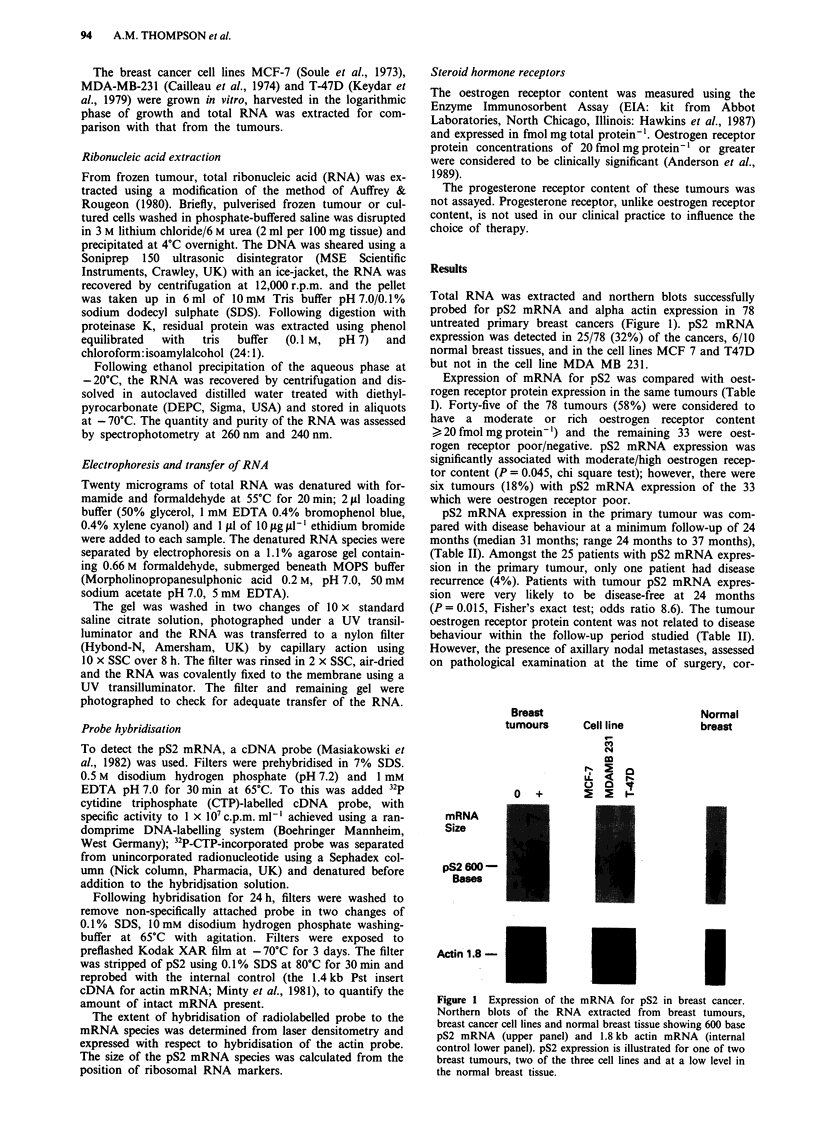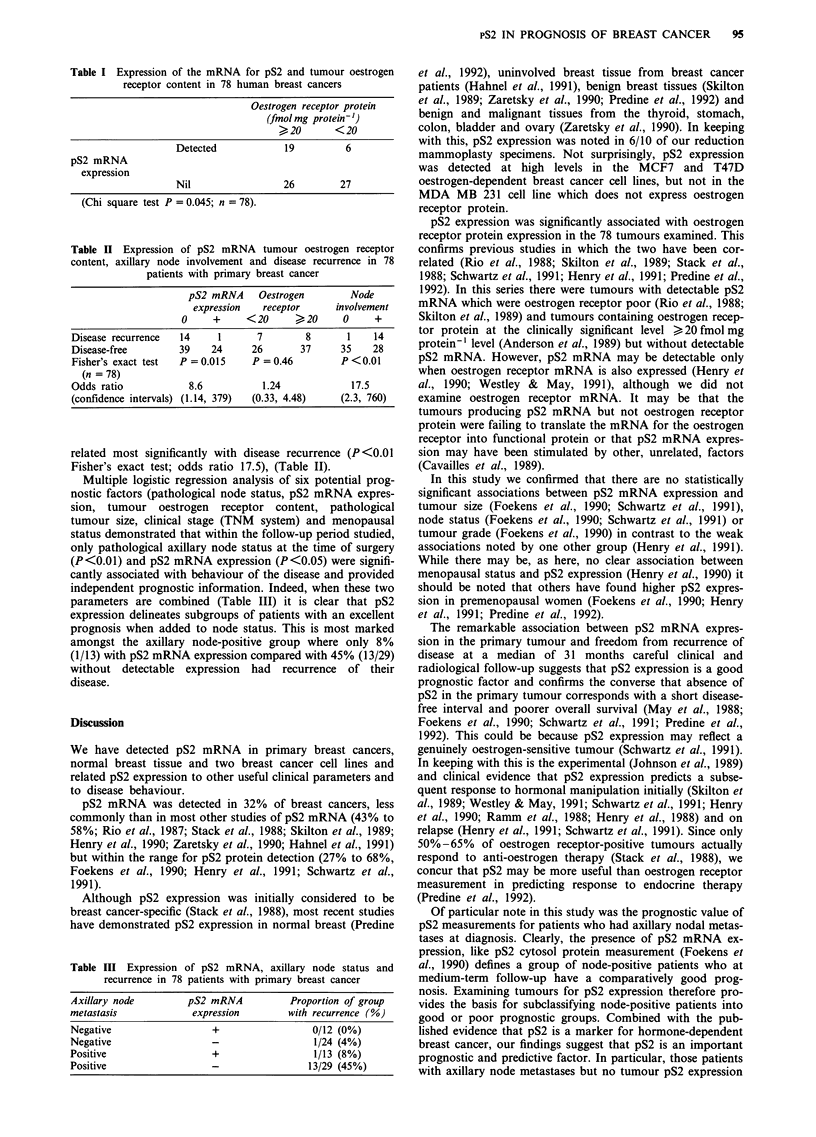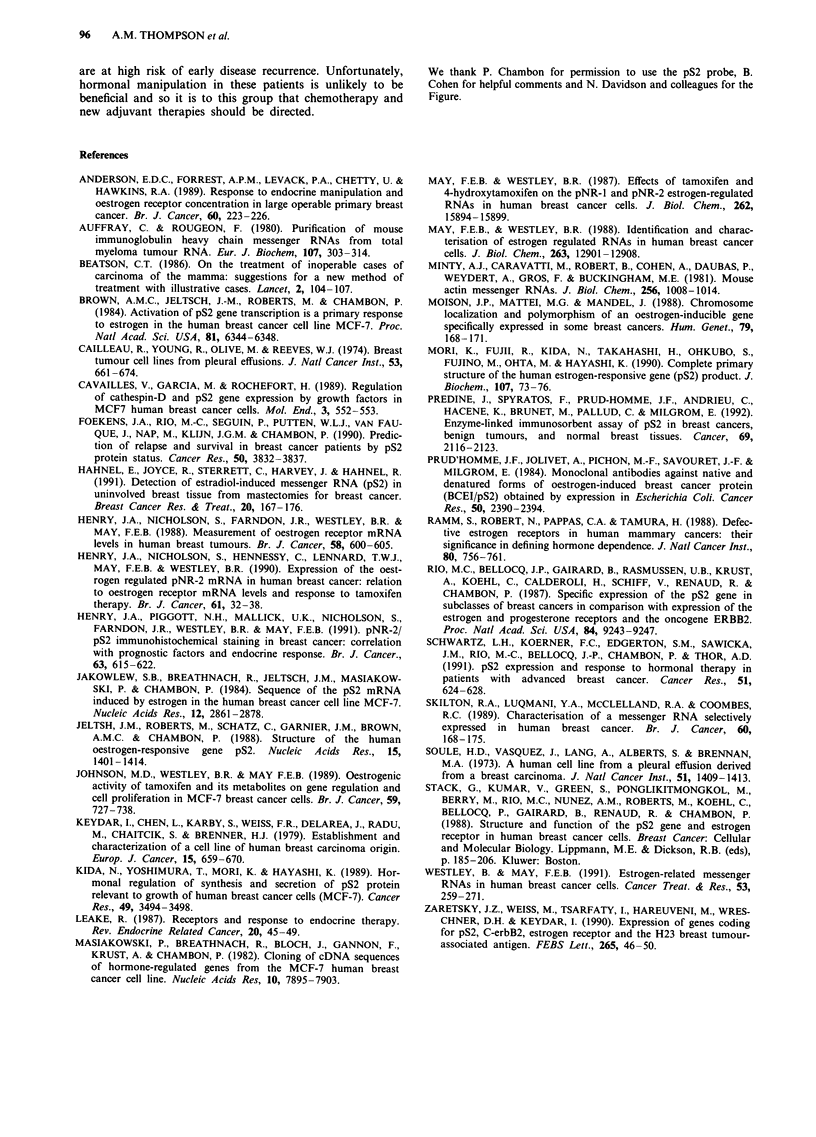# pS2 is an independent factor of good prognosis in primary breast cancer.

**DOI:** 10.1038/bjc.1993.292

**Published:** 1993-07

**Authors:** A. M. Thompson, R. A. Hawkins, R. A. Elton, C. M. Steel, U. Chetty, D. C. Carter

**Affiliations:** University Department of Surgery, Royal Infirmary, Edinburgh, UK.

## Abstract

**Images:**


Br. J. Cancer (1993), 68, 93-96 © Macmillan Press Ltd., 1993

### pS2 is an independent factor of good prognosis in primary breast cancer

A.M. Thompson[1], R.A. Hawkins[1], R.A. Elton[2], C.M. Steel[3], U. Chetty[1] & D.C. Carter[1]

[1]*University Department of Surgery, Royal Infirmary, Lauriston Place, Edinburgh, EH3 9YW;* [2]*Medical Statistics Unit, University of Edinburgh, Teviot Place, Edinburgh, EH8 9AG;* [3]*MRC Human Genetics Unit, Western General Hospital, Crewe Road, Edinburgh, EH4 2XU, UK.*

**Summary** In breast cancer, oestrogen regulated genes, such as pS2, may be expressed in well differentiated tumours with a good prognosis. We have examined pS2 mRNA expression in 78 primary, untreated breast cancers and related pS2 expression to disease behaviour and known prognostic factors. pS2 mRNA expression was detected in 25/78 (32%) of cancers and was significantly associated with a moderate/high oestrogen receptor content ($P = 0.045$, Chi Square test). pS2 mRNA expression was associated with freedom from disease at median 31 months clinical and radiological follow-up ($P = 0.015$, Fisher's exact test, odds ratio 8.6).

Using multiple logistic regression analysis of six potential prognostic factors only pathological axillary node status ($P < 0.01$) and pS2 mRNA expression ($P < 0.05$) provided independent prognostic information. Furthermore, pS2 was associated with a good prognosis in the axillary node positive patients where only 1/13 (8%) with pS2 mRNA expression compared with 13/29 (45%) without detectable expression had recurrence of their disease. These data provides strong support for pS2 as a useful independent prognostic factor in primary breast cancer.

It is almost 100 years since the oestrogen-dependence of breast cancer was first demonstrated (Beatson, 1896). However, since the oestrogen receptor content of a breast tumour is an imperfect predictor of response to endocrine therapy (Leake, 1987), with defects in the regulation of oestrogen receptor function perhaps explaining why some patients fail to respond (Schwartz *et al.*, 1991), attention has turned to oestrogen-regulated genes in an attempt to define more accurately the role of oestrogen in individual tumours. One of these genes, variously known as pS2 (Masiakowski *et al.*, 1982), pNR2 (Westley & May, 1991), Md2 (Skilton *et al.*, 1989) and BCEI (Prud, homme *et al.*, 1984) has received particular attention.

Located on chromosome 21q (Moison *et al.*, 1988), the pS2 gene comprises 3 exons of 125, 153 and 212 base-pairs interrupted by 2 introns of 3.1 kb (intron A) and 0.77 kb (intron B) (Jakowlew *et al.*, 1984; Jeltsh *et al.*, 1988; Stack *et al.*, 1988; Mori *et al.*, 1990). There are two start sites for transcription, one of which predominates (Mori *et al.*, 1990) to generate a 600-base mRNA (Masiakowski *et al.*, 1982; Stack *et al.*, 1988) that encodes an 84-amino-acid, precursor protein of 9.14 kda (Stack *et al.*, 1988), which is cleaved to a 7 kda 60-amino-acid polypeptide (Stack *et al.*, 1988, Mori *et al.*, 1990) and is secreted from the cell (Brown *et al.*, 1984). This cysteine-rich protein (Rio *et al.*, 1987), capable of forming 3 disulphide bonds (Stack *et al.*, 1988), has structural similarities to Insulin Like Growth Factor I and Insulin Like Growth Factor II (Rio *et al.*, 1987; Stack *et al.*, 1988) and porcine pancreatic spasmolytic polypeptide (Rio *et al.*, 1988).

Expression of the mRNA for pS2 increases in response to an oestrogenic stimulus (Masiakowski *et al.*, 1982; Brown *et al.*, 1984; Stack *et al.*, 1988) and coincides with the appearance of highly associated oestrogen to oestrogen-receptor complexes in the nucleus (Brown *et al.*, 1984). Oestrogenic stimulation of pS2 can be antagonised by tamoxifen (May & Westley, 1987) although tamoxifen may itself act as a weak agonist for pS2 expression (Johnston *et al.*, 1989). While pS2 expression is not influenced by progestins, glucocorticoids or androgens (Brown *et al.*, 1984), pS2 can be induced by EGF and by agents elevating cAMP through indirect mechanisms (requiring protein synthesis) in addition to the primary transcriptional effects of oestrogenic stimulation (Cavailles *et al.*, 1989).

However, the function of pS2 and its biological significance remain uncertain. pS2 is certainly secreted from oestrogen-dependent cell lines (Brown *et al.*, 1984) and does not appear to stimulate DNA synthesis directly (Kida *et al.*, 1989) but may act in an autocrine or paracrine manner. In view of its oestrogen-dependence, pS2 expression may reflect tumour differentiation and therefore may be an index of prognosis.

The aims of this study were to examine pS2 mRNA expression in primary breast cancer and to determine whether pS2 could be related to patient prognosis and to other factors of established prognostic significance.

### Materials and methods

### *Patients*

Seventy eight female patients with primary breast cancer attending the Breast Unit, University Department of Surgery, Edinburgh were studied. At the time of diagnosis the patients (age range 34-84) had received no anticancer therapy and had no evidence of distant metastatic disease. Twenty-six of the women were premenopausal and the remaining 52 postmenopausal. The primary tumour was less than 5 cm ($T_2$) in 44 patients, and over 5 cm ($T_3$) or locally advanced ($T_4$) in 34 patients. There was histological evidence of nodal metastasis in 42 of the 78 patients.

Patient follow-up was conducted at 3 to 4 month intervals for the first 24 months and thereafter at 6 month intervals. In addition to clinical examination, annual chest radiography and, where appropriate, mammography, were performed. On disease relapse, patients were restaged to establish the extent of disease and the sites of recurrence.

### *Tissues*

Tumour tissue was snap frozen in liquid nitrogen at the time of surgery and stored at −70°C. Adjacent tissue was submitted for the determination of oestrogen receptor content and for histopathological confirmation of malignancy (although this did not include pathological grade).

Normal breast tissue from ten patients who underwent reduction mammoplasty and who had no personal or family history of breast cancer were also obtained fresh and snap frozen.

Correspondence: A.M. Thompson, University Department of Surgery, Royal Infirmary, Lauriston Place, Edinburgh, UK. Received 21 October 1992; and in revised form 5 January 1993.

The breast cancer cell lines MCF-7 (Soule et al., 1973), MDA-MB-231 (Cailleau et al., 1974) and T-47D (Keydar et al., 1979) were grown *in vitro*, harvested in the logarithmic phase of growth and total RNA was extracted for comparison with that from the tumours.

*Ribonucleic acid extraction*

From frozen tumour, total ribonucleic acid (RNA) was extracted using a modification of the method of Auffrey & Rougeon (1980). Briefly, pulverised frozen tumour or cultured cells washed in phosphate-buffered saline was disrupted in 3 M lithium chloride/6 M urea (2 ml per 100 mg tissue) and precipitated at 4°C overnight. The DNA was sheared using a Soniprep 150 ultrasonic disintegrator (MSE Scientific Instruments, Crawley, UK) with an ice-jacket, the RNA was recovered by centrifugation at 12,000 r.p.m. and the pellet was taken up in 6 ml of 10 mM Tris buffer pH 7.0/0.1% sodium dodecyl sulphate (SDS). Following digestion with proteinase K, residual protein was extracted using phenol equilibrated with tris buffer (0.1 M, pH 7) and chloroform:isoamylalcohol (24:1).

Following ethanol precipitation of the aqueous phase at −20°C, the RNA was recovered by centrifugation and dissolved in autoclaved distilled water treated with diethylpyrocarbonate (DEPC, Sigma, USA) and stored in aliquots at −70°C. The quantity and purity of the RNA was assessed by spectrophotometry at 260 nm and 240 nm.

*Electrophoresis and transfer of RNA*

Twenty micrograms of total RNA was denatured with formamide and formaldehyde at 55°C for 20 min; 2 μl loading buffer (50% glycerol, 1 mM EDTA 0.4% bromophenol blue, 0.4% xylene cyanol) and 1 μl of 10 $\mu g \mu l^{-1}$ ethidium bromide were added to each sample. The denatured RNA species were separated by electrophoresis on a 1.1% agarose gel containing 0.66 M formaldehyde, submerged beneath MOPS buffer (Morpholinopropanesulphonic acid 0.2 M, pH 7.0, 50 mM sodium acetate pH 7.0, 5 mM EDTA).

The gel was washed in two changes of 10 × standard saline citrate solution, photographed under a UV transilluminator and the RNA was transferred to a nylon filter (Hybond-N, Amersham, UK) by capillary action using 10 × SSC over 8 h. The filter was rinsed in 2 × SSC, air-dried and the RNA was covalently fixed to the membrane using a UV transilluminator. The filter and remaining gel were photographed to check for adequate transfer of the RNA.

*Probe hybridisation*

To detect the pS2 mRNA, a cDNA probe (Masiakowski et al., 1982) was used. Filters were prehybridised in 7% SDS. 0.5 M disodium hydrogen phosphate (pH 7.2) and 1 mM EDTA pH 7.0 for 30 min at 65°C. To this was added $^{32}P$ cytidine triphosphate (CTP)-labelled cDNA probe, with specific activity to $1 \times 10^7$ c.p.m. $ml^{-1}$ achieved using a randomprime DNA-labelling system (Boehringer Mannheim, West Germany); $^{32}P$-CTP-incorporated probe was separated from unincorporated radionucleotide using a Sephadex column (Nick column, Pharmacia, UK) and denatured before addition to the hybridisation solution.

Following hybridisation for 24 h, filters were washed to remove non-specifically attached probe in two changes of 0.1% SDS, 10 mM disodium hydrogen phosphate washing-buffer at 65°C with agitation. Filters were exposed to preflashed Kodak XAR film at −70°C for 3 days. The filter was stripped of pS2 using 0.1% SDS at 80°C for 30 min and reprobed with the internal control (the 1.4 kb Pst insert cDNA for actin mRNA; Minty et al., 1981), to quantify the amount of intact mRNA present.

The extent of hybridisation of radiolabelled probe to the mRNA species was determined from laser densitometry and expressed with respect to hybridisation of the actin probe. The size of the pS2 mRNA species was calculated from the position of ribosomal RNA markers.

*Steroid hormone receptors*

The oestrogen receptor content was measured using the Enzyme Immunosorbent Assay (EIA: kit from Abbot Laboratories, North Chicago, Illinois: Hawkins et al., 1987) and expressed in fmol mg total protein$^{-1}$. Oestrogen receptor protein concentrations of 20 fmol mg protein$^{-1}$ or greater were considered to be clinically significant (Anderson et al., 1989).

The progesterone receptor content of these tumours was not assayed. Progesterone receptor, unlike oestrogen receptor content, is not used in our clinical practice to influence the choice of therapy.

### Results

Total RNA was extracted and northern blots successfully probed for pS2 mRNA and alpha actin expression in 78 untreated primary breast cancers (Figure 1). pS2 mRNA expression was detected in 25/78 (32%) of the cancers, 6/10 normal breast tissues, and in the cell lines MCF 7 and T47D but not in the cell line MDA MB 231.

Expression of mRNA for pS2 was compared with oestrogen receptor protein expression in the same tumours (Table I). Forty-five of the 78 tumours (58%) were considered to have a moderate or rich oestrogen receptor content ⩾20 fmol mg protein$^{-1}$) and the remaining 33 were oestrogen receptor poor/negative. pS2 mRNA expression was significantly associated with moderate/high oestrogen receptor content ($P = 0.045$, chi square test); however, there were six tumours (18%) with pS2 mRNA expression of the 33 which were oestrogen receptor poor.

pS2 mRNA expression in the primary tumour was compared with disease behaviour at a minimum follow-up of 24 months (median 31 months; range 24 months to 37 months), (Table II). Amongst the 25 patients with pS2 mRNA expression in the primary tumour, only one patient had disease recurrence (4%). Patients with tumour pS2 mRNA expression were very likely to be disease-free at 24 months ($P = 0.015$, Fisher's exact test; odds ratio 8.6). The tumour oestrogen receptor protein content was not related to disease behaviour within the follow-up period studied (Table II). However, the presence of axillary nodal metastases, assessed on pathological examination at the time of surgery, cor-

**Figure 1** Expression of the mRNA for pS2 in breast cancer. Northern blots of the RNA extracted from breast tumours, breast cancer cell lines and normal breast tissue showing 600 base pS2 mRNA (upper panel) and 1.8 kb actin mRNA (internal control lower panel). pS2 expression is illustrated for one of two breast tumours, two of the three cell lines and at a low level in the normal breast tissue.

**Table I** Expression of the mRNA for pS2 and tumour oestrogen receptor content in 78 human breast cancers

| | | *Oestrogen receptor protein (fmol mg protein$^{-1}$)* | |
|---|---|---|---|
| | | *≥20* | *<20* |
| pS2 mRNA expression | Detected | 19 | 6 |
| | Nil | 26 | 27 |

(Chi square test $P = 0.045$; $n = 78$).

**Table II** Expression of pS2 mRNA tumour oestrogen receptor content, axillary node involvement and disease recurrence in 78 patients with primary breast cancer

| | *pS2 mRNA expression* | | *Oestrogen receptor* | | *Node involvement* | |
|---|---|---|---|---|---|---|
| | *0* | *+* | *<20* | *≥20* | *0* | *+* |
| Disease recurrence | 14 | 1 | 7 | 8 | 1 | 14 |
| Disease-free | 39 | 24 | 26 | 37 | 35 | 28 |
| Fisher's exact test ($n = 78$) | $P = 0.015$ | | $P = 0.46$ | | $P < 0.01$ | |
| Odds ratio | 8.6 | | 1.24 | | 17.5 | |
| (confidence intervals) | (1.14, 379) | | (0.33, 4.48) | | (2.3, 760) | |

related most significantly with disease recurrence ($P<0.01$ Fisher's exact test; odds ratio 17.5), (Table II).

Multiple logistic regression analysis of six potential prognostic factors (pathological node status, pS2 mRNA expression, tumour oestrogen receptor content, pathological tumour size, clinical stage (TNM system) and menopausal status demonstrated that within the follow-up period studied, only pathological axillary node status at the time of surgery ($P<0.01$) and pS2 mRNA expression ($P<0.05$) were significantly associated with behaviour of the disease and provided independent prognostic information. Indeed, when these two parameters are combined (Table III) it is clear that pS2 expression delineates subgroups of patients with an excellent prognosis when added to node status. This is most marked amongst the axillary node-positive group where only 8% (1/13) with pS2 mRNA expression compared with 45% (13/29) without detectable expression had recurrence of their disease.

### Discussion

We have detected pS2 mRNA in primary breast cancers, normal breast tissue and two breast cancer cell lines and related pS2 expression to other useful clinical parameters and to disease behaviour.

pS2 mRNA was detected in 32% of breast cancers, less commonly than in most other studies of pS2 mRNA (43% to 58%; Rio *et al.*, 1987; Stack *et al.*, 1988; Skilton *et al.*, 1989; Henry *et al.*, 1990; Zaretsky *et al.*, 1990; Hahnel *et al.*, 1991) but within the range for pS2 protein detection (27% to 68%, Foekens *et al.*, 1990; Henry *et al.*, 1991; Schwartz *et al.*, 1991).

Although pS2 expression was initially considered to be breast cancer-specific (Stack *et al.*, 1988), most recent studies have demonstrated pS2 expression in normal breast (Predine *et al.*, 1992), uninvolved breast tissue from breast cancer patients (Hahnel *et al.*, 1991), benign breast tissues (Skilton *et al.*, 1989; Zaretsky *et al.*, 1990; Predine *et al.*, 1992) and benign and malignant tissues from the thyroid, stomach, colon, bladder and ovary (Zaretsky *et al.*, 1990). In keeping with this, pS2 expression was noted in 6/10 of our reduction mammoplasty specimens. Not surprisingly, pS2 expression was detected at high levels in the MCF7 and T47D oestrogen-dependent breast cancer cell lines, but not in the MDA MB 231 cell line which does not express oestrogen receptor protein.

pS2 expression was significantly associated with oestrogen receptor protein expression in the 78 tumours examined. This confirms previous studies in which the two have been correlated (Rio *et al.*, 1988; Skilton *et al.*, 1989; Stack *et al.*, 1988; Schwartz *et al.*, 1991; Henry *et al.*, 1991; Predine *et al.*, 1992). In this series there were tumours with detectable pS2 mRNA which were oestrogen receptor poor (Rio *et al.*, 1988; Skilton *et al.*, 1989) and tumours containing oestrogen receptor protein at the clinically significant level ≥20 fmol mg protein$^{-1}$ level (Anderson *et al.*, 1989) but without detectable pS2 mRNA. However, pS2 mRNA may be detectable only when oestrogen receptor mRNA is also expressed (Henry *et al.*, 1990; Westley & May, 1991), although we did not examine oestrogen receptor mRNA. It may be that the tumours producing pS2 mRNA but not oestrogen receptor protein were failing to translate the mRNA for the oestrogen receptor into functional protein or that pS2 mRNA expression may have been stimulated by other, unrelated, factors (Cavailles *et al.*, 1989).

In this study we confirmed that there are no statistically significant associations between pS2 mRNA expression and tumour size (Foekens *et al.*, 1990; Schwartz *et al.*, 1991), node status (Foekens *et al.*, 1990; Schwartz *et al.*, 1991) or tumour grade (Foekens *et al.*, 1990) in contrast to the weak associations noted by one other group (Henry *et al.*, 1991). While there may be, as here, no clear association between menopausal status and pS2 expression (Henry *et al.*, 1990) it should be noted that others have found higher pS2 expression in premenopausal women (Foekens *et al.*, 1990; Henry *et al.*, 1991; Predine *et al.*, 1992).

The remarkable association between pS2 mRNA expression in the primary tumour and freedom from recurrence of disease at a median of 31 months careful clinical and radiological follow-up suggests that pS2 expression is a good prognostic factor and confirms the converse that absence of pS2 in the primary tumour corresponds with a short disease-free interval and poorer overall survival (May *et al.*, 1988; Foekens *et al.*, 1990; Schwartz *et al.*, 1991; Predine *et al.*, 1992). This could be because pS2 expression may reflect a genuinely oestrogen-sensitive tumour (Schwartz *et al.*, 1991). In keeping with this is the experimental (Johnson *et al.*, 1989) and clinical evidence that pS2 expression predicts a subsequent response to hormonal manipulation initially (Skilton *et al.*, 1989; Westley & May, 1991; Schwartz *et al.*, 1991; Henry *et al.*, 1990; Ramm *et al.*, 1988; Henry *et al.*, 1988) and on relapse (Henry *et al.*, 1991; Schwartz *et al.*, 1991). Since only 50%–65% of oestrogen receptor-positive tumours actually respond to anti-oestrogen therapy (Stack *et al.*, 1988), we concur that pS2 may be more useful than oestrogen receptor measurement in predicting response to endocrine therapy (Predine *et al.*, 1992).

Of particular note in this study was the prognostic value of pS2 measurements for patients who had axillary nodal metastases at diagnosis. Clearly, the presence of pS2 mRNA expression, like pS2 cytosol protein measurement (Foekens *et al.*, 1990) defines a group of node-positive patients who at medium-term follow-up have a comparatively good prognosis. Examining tumours for pS2 expression therefore provides the basis for subclassifying node-positive patients into good or poor prognostic groups. Combined with the published evidence that pS2 is a marker for hormone-dependent breast cancer, our findings suggest that pS2 is an important prognostic and predictive factor. In particular, those patients with axillary node metastases but no tumour pS2 expression

**Table III** Expression of pS2 mRNA, axillary node status and recurrence in 78 patients with primary breast cancer

| *Axillary node metastasis* | *pS2 mRNA expression* | *Proportion of group with recurrence (%)* |
|---|---|---|
| Negative | + | 0/12 (0%) |
| Negative | – | 1/24 (4%) |
| Positive | + | 1/13 (8%) |
| Positive | – | 13/29 (45%) |

are at high risk of early disease recurrence. Unfortunately, hormonal manipulation in these patients is unlikely to be beneficial and so it is to this group that chemotherapy and new adjuvant therapies should be directed.

We thank P. Chambon for permission to use the pS2 probe, B. Cohen for helpful comments and N. Davidson and colleagues for the Figure.

### References

ANDERSON, E.D.C., FORREST, A.P.M., LEVACK, P.A., CHETTY, U. & HAWKINS, R.A. (1989). Response to endocrine manipulation and oestrogen receptor concentration in large operable primary breast cancer. *Br. J. Cancer*, **60**, 223-226.

AUFFRAY, C. & ROUGEON, F. (1980). Purification of mouse immunoglobulin heavy chain messenger RNAs from total myeloma tumour RNA. *Eur. J. Biochem*, **107**, 303-314.

BEATSON, C.T. (1986). On the treatment of inoperable cases of carcinoma of the mamma: suggestions for a new method of treatment with illustrative cases. *Lancet*, **2**, 104-107.

BROWN, A.M.C., JELTSCH, J.-M., ROBERTS, M. & CHAMBON, P. (1984). Activation of pS2 gene transcription is a primary response to estrogen in the human breast cancer cell line MCF-7. *Proc. Natl Acad. Sci. USA*, **81**, 6344-6348.

CAILLEAU, R., YOUNG, R., OLIVE, M. & REEVES, W.J. (1974). Breast tumour cell lines from pleural effusions. *J. Natl Cancer Inst.*, **53**, 661-674.

CAVAILLES, V., GARCIA, M. & ROCHEFORT, H. (1989). Regulation of cathespin-D and pS2 gene expression by growth factors in MCF7 human breast cancer cells. *Mol. End.*, **3**, 552-553.

FOEKENS, J.A., RIO, M.-C., SEGUIN, P., PUTTEN, W.L.J., VAN FAUQUE, J., NAP, M., KLIJN, J.G.M. & CHAMBON, P. (1990). Prediction of relapse and survival in breast cancer patients by pS2 protein status. *Cancer Res.*, **50**, 3832-3837.

HAHNEL, E., JOYCE, R., STERRETT, C., HARVEY, J. & HAHNEL, R. (1991). Detection of estradiol-induced messenger RNA (pS2) in uninvolved breast tissue from mastectomies for breast cancer. *Breast Cancer Res. & Treat.*, **20**, 167-176.

HENRY, J.A., NICHOLSON, S., FARNDON, J.R., WESTLEY, B.R. & MAY, F.E.B. (1988). Measurement of oestrogen receptor mRNA levels in human breast tumours. *Br. J. Cancer*, **58**, 600-605.

HENRY, J.A., NICHOLSON, S., HENNESSY, C., LENNARD, T.W.J., MAY, F.E.B. & WESTLEY, B.R. (1990). Expression of the oestrogen regulated pNR-2 mRNA in human breast cancer: relation to oestrogen receptor mRNA levels and response to tamoxifen therapy. *Br. J. Cancer*, **61**, 32-38.

HENRY, J.A., PIGGOTT, N.H., MALLICK, U.K., NICHOLSON, S., FARNDON, J.R., WESTLEY, B.R. & MAY, F.E.B. (1991). pNR-2/pS2 immunohistochemical staining in breast cancer: correlation with prognostic factors and endocrine response. *Br. J. Cancer.*, **63**, 615-622.

JAKOWLEW, S.B., BREATHNACH, R., JELTSCH, J.M., MASIAKOWSKI, P. & CHAMBON, P. (1984). Sequence of the pS2 mRNA induced by estrogen in the human breast cancer cell line MCF-7. *Nucleic Acids Res.*, **12**, 2861-2878.

JELTSH, J.M., ROBERTS, M., SCHATZ, C., GARNIER, J.M., BROWN, A.M.C. & CHAMBON, P. (1988). Structure of the human oestrogen-responsive gene pS2. *Nucleic Acids Res.*, **15**, 1401-1414.

JOHNSON, M.D., WESTLEY, B.R. & MAY F.E.B. (1989). Oestrogenic activity of tamoxifen and its metabolites on gene regulation and cell proliferation in MCF-7 breast cancer cells. *Br. J. Cancer*, **59**, 727-738.

KEYDAR, I., CHEN, L., KARBY, S., WEISS, F.R., DELAREA, J., RADU, M., CHAITCIK, S. & BRENNER, H.J. (1979). Establishment and characterization of a cell line of human breast carcinoma origin. *Europ. J. Cancer*, **15**, 659-670.

KIDA, N., YOSHIMURA, T., MORI, K. & HAYASHI, K. (1989). Hormonal regulation of synthesis and secretion of pS2 protein relevant to growth of human breast cancer cells (MCF-7). *Cancer Res.*, **49**, 3494-3498.

LEAKE, R. (1987). Receptors and response to endocrine therapy. *Rev. Endocrine Related Cancer*, **20**, 45-49.

MASIAKOWSKI, P., BREATHNACH, R., BLOCH, J., GANNON, F., KRUST, A. & CHAMBON, P. (1982). Cloning of cDNA sequences of hormone-regulated genes from the MCF-7 human breast cancer cell line. *Nucleic Acids Res*, **10**, 7895-7903.

MAY, F.E.B. & WESTLEY, B.R. (1987). Effects of tamoxifen and 4-hydroxytamoxifen on the pNR-1 and pNR-2 estrogen-regulated RNAs in human breast cancer cells. *J. Biol. Chem.*, **262**, 15894-15899.

MAY, F.E.B., & WESTLEY, B.R. (1988). Identification and characterisation of estrogen regulated RNAs in human breast cancer cells. *J. Biol. Chem.*, **263**, 12901-12908.

MINTY, A.J., CARAVATTI, M., ROBERT, B., COHEN, A., DAUBAS, P., WEYDERT, A., GROS, F. & BUCKINGHAM, M.E. (1981). Mouse actin messenger RNAs. *J. Biol. Chem.*, **256**, 1008-1014.

MOISON, J.P., MATTEI, M.G. & MANDEL, J. (1988). Chromosome localization and polymorphism of an oestrogen-inducible gene specifically expressed in some breast cancers. *Hum. Genet.*, **79**, 168-171.

MORI, K., FUJII, R., KIDA, N., TAKAHASHI, H., OHKUBO, S., FUJINO, M., OHTA, M. & HAYASHI, K. (1990). Complete primary structure of the human estrogen-responsive gene (pS2) product. *J. Biochem.*, **107**, 73-76.

PREDINE, J., SPYRATOS, F., PRUD-HOMME, J.F., ANDRIEU, C., HACENE, K., BRUNET, M., PALLUD, C. & MILGROM, E. (1992). Enzyme-linked immunosorbent assay of pS2 in breast cancers, benign tumours, and normal breast tissues. *Cancer*, **69**, 2116-2123.

PRUD'HOMME, J.F., JOLIVET, A., PICHON, M.-F., SAVOURET, J.-F. & MILGROM, E. (1984). Monoclonal antibodies against native and denatured forms of oestrogen-induced breast cancer protein (BCEI/pS2) obtained by expression in *Escherichia Coli. Cancer Res.*, **50**, 2390-2394.

RAMM, S., ROBERT, N., PAPPAS, C.A. & TAMURA, H. (1988). Defective estrogen receptors in human mammary cancers: their significance in defining hormone dependence. *J. Natl Cancer Inst.*, **80**, 756-761.

RIO, M.C., BELLOCQ, J.P., GAIRARD, B., RASMUSSEN, U.B., KRUST, A., KOEHL, C., CALDEROLI, H., SCHIFF, V., RENAUD, R. & CHAMBON, P. (1987). Specific expression of the pS2 gene in subclasses of breast cancers in comparison with expression of the estrogen and progesterone receptors and the oncogene ERBB2. *Proc. Natl Acad. Sci. USA*, **84**, 9243-9247.

SCHWARTZ, L.H., KOERNER, F.C., EDGERTON, S.M., SAWICKA, J.M., RIO, M.-C., BELLOCQ, J.-P., CHAMBON, P. & THOR, A.D. (1991). pS2 expression and response to hormonal therapy in patients with advanced breast cancer. *Cancer Res.*, **51**, 624-628.

SKILTON, R.A., LUQMANI, Y.A., McCLELLAND, R.A. & COOMBES, R.C. (1989). Characterisation of a messenger RNA selectively expressed in human breast cancer. *Br. J. Cancer*, **60**, 168-175.

SOULE, H.D., VASQUEZ, J., LANG, A., ALBERTS, S. & BRENNAN, M.A. (1973). A human cell line from a pleural effusion derived from a breast carcinoma. *J. Natl Cancer Inst.*, **51**, 1409-1413.

STACK, G., KUMAR, V., GREEN, S., PONGLIKITMONGKOL, M., BERRY, M., RIO, M.C., NUNEZ, A.M., ROBERTS, M., KOEHL, C., BELLOCQ, P., GAIRARD, B., RENAUD, R. & CHAMBON, P. (1988). Structure and function of the pS2 gene and estrogen receptor in human breast cancer cells. *Breast Cancer*: Cellular and Molecular Biology. Lippmann, M.E. & Dickson, R.B. (eds), p. 185-206. Kluwer: Boston.

WESTLEY, B. & MAY, F.E.B. (1991). Estrogen-related messenger RNAs in human breast cancer cells. *Cancer Treat. & Res.*, **53**, 259-271.

ZARETSKY, J.Z., WEISS, M., TSARFATY, I., HAREUVENI, M., WRESCHNER, D.H. & KEYDAR, I. (1990). Expression of genes coding for pS2, C-erbB2, estrogen receptor and the H23 breast tumour-associated antigen. *FEBS Lett.*, **265**, 46-50.